# Effect of repetitive transcranial magnetic stimulation on upper limb motor function in stroke patients with right hemiplegia based on EEG microstates and EMG

**DOI:** 10.3389/fneur.2025.1587928

**Published:** 2025-09-29

**Authors:** Xianxian Yu, Rong Xin, Siman Cheng, Jiale Xie, Gengqiang Ling, Xin Wei, Pu Wang

**Affiliations:** ^1^Department of Rahabilitation Medicine, The Seventh Affiliated Hospital of Sun Yat-Sen University, Shenzhen, China; ^2^Expert Workstation in Sichuan Province, Chengdu Jincheng University, Chengdu, China; ^3^School of Software, Xi’an Jiaotong University, Xi'an, China; ^4^Guangdong Engineering and Technology Research Center for Rehabilitation Medicine and Translation, Guangzhou, China

**Keywords:** rTMS (repetitive transcranial magnetic stimulation), stroke, microstates, EEG, EMG (electromyogram)

## Abstract

**Introduction:**

Stroke severely impairs neural function and daily living, creating an urgent need for innovative rehabilitation strategies. This study aimed to investigate the effects of transcranial magnetic stimulation (TMS) on upper limb motor recovery in stroke patients, combining EEG microstate analysis and EMG to elucidate associated neuromuscular and cortical changes.

**Methods:**

Twenty patients with right-hemiplegic stroke and twenty healthy controls were enrolled. Patients underwent Fugl-Meyer Assessment for Upper Extremity (FMA-UE) and Action Research Arm Test (ARAT) before and after repetitive TMS (rTMS) intervention. Resting-state EEG and EMG recordings were acquired pre- and post-one week of rTMS treatment.

**Results:**

Following rTMS, patients exhibited significant improvements in FMA-UE and ARAT scores (*p* < 0.05). EEG microstate analysis indicated that stroke patients initially showed decreased time coverage and occurrence of Microstate B (associated with sensorimotor integration, *p* < 0.05). After rTMS, these parameters increased markedly, approaching levels observed in healthy controls (*p* < 0.05). In contrast, Microstate C (motor execution-related) and Microstate D (attention-related) displayed reduced duration and coverage post-intervention (*p* < 0.05). Critically, enhancement in Microstate B metrics correlated with improved motor coordination in specific muscles (flexor/extensor carpi ulnaris, *p* < 0.05), while changes in Microstate C were positively correlated with gains in upper limb strength.

**Discussion:**

These findings highlight two central mechanisms: (1) rTMS promotes motor recovery in hemiplegic patients by normalizing cortical dynamics, as reflected in microstate reorganization; (2) Microstate B and C represent promising neurophysiological biomarkers for tracking rehabilitation progress, with the former reflecting motor coordination and the latter indexing strength recovery. This study bridges microstate-level neurophysiological changes and functional improvements, supporting rTMS as a precision intervention in stroke neurorehabilitation. Further research should validate these biomarkers in larger cohorts and explore microstate-guided rTMS protocols.

**Clinical trial registration:**

chictr.org.cn, Identifier: ChiCTR2100049509.

## 1 Introduction

Stroke is the second leading global cause of disability and mortality (1), a serious neurological disorder associated with determinants of motor dysfunction (2). The subsequent neurological deficits have profound implications for the quality of life of patients, with approximately 50% of survivors experiencing long-term disability (3, 4). Among stroke survivors, limb movement disorders, especially in the upper limbs, are common, with limited recovery prospects (5). Thus, effective rehabilitation programs for upper limb motor function recovery in hemiplegic stroke patients are essential to alleviate these burdens.

Stroke is classified into hemorrhagic and ischemic types, with ischemic stroke constituting around 85% of cases, typically arising from vascular blockages leading to focal neural dysfunction in specific brain regions (6). To address this neural dysfunction, neuromodulation techniques that can directly target and alter cortical excitability are required. Inducing neurofunctional changes in the affected brain areas through the stimulation of peripheral nerves is a commonly used treatment method (7). Stroke patients typically exhibit upper limb motor impairments characterized by abnormal muscle tone and altered muscle synergy patterns (8). Barker et al. (9) first reported a non-invasive transcranial magnetic stimulation technique in 1985. The principle is to generate a pulsed magnetic field through the high-intensity current circulating inside the coil placed on the scalp, and according to Faraday’s principle of electromagnetic induction, the magnetic field generates an induced electric field in the cerebral cortex, which directly or indirectly depolarizes the interneurons through the axons and generates an action potential, i.e., neuronal discharge. Such discharges can affect the excitability of neurons, which in turn affects their synaptic transmission and neural network activity, influences metabolism and related electrophysiological activities in the brain (10), and regulates and intervenes in brain function (11). Repetitive transcranial magnetic stimulation (rTMS) is a technology developed based on transcranial magnetic stimulation, which can issue repetitive, non-attenuating, and regular pulse waves to continuously stimulate the cerebral cortex, which is painless, non-invasive, safe, reliable, and easy to operate. It can be used to study the functional connectivity of the brain, cortical plasticity, and the pathological mechanisms of neurological diseases. Repetitive transcranial magnetic stimulation is now widely used in the treatment of movement disorders and the rehabilitation of stroke patients (12, 13). The therapeutic potential of rTMS stems from its ability to modulate cortical excitability and induce neuroplasticity. These neurophysiological effects translate into meaningful functional improvements, as evidenced by empirical studies showing substantial augmentation in muscle strength, enhanced coordination, and greater overall motor capacity (14). The efficacy of rTMS in promoting upper limb motor recovery post-stroke is further supported by recent comprehensive reviews of the clinical literature (15, 16). Studying upper limb motor impairments and assessing the effectiveness of rehabilitation interventions require close monitoring of changes in muscle, nerves, and motor function. Presently, clinical assessments predominantly depend on scales like the Fugl-Meyer Assessment (FMA) (17). However, these qualitative assessment methods rely on behavioral scores from specific motor tasks, which can be influenced by subjectivity. Furthermore, they often lack insights into the underlying mechanisms involving neural factors and motor control. As a result, there is a clinical need for quantitative assessment methods incorporating physiological data.

Scalp electroencephalogram (EEG) signals convey information regarding the brain’s control of limb movement (18, 19). Electromyography (EMG) signals primarily reflect efferent motor commands from the central nervous system to muscles, capturing compound muscle action potentials generated by the activation of motor units (20). The motor nervous system transmits control information through oscillatory activity, which can mirror the coupling of cortical and muscle functions. Research has shown a strong correlation between surface Electromyogram (sEMG) and muscle activity states and functional conditions, making it suitable for assessing muscle activation patterns, coordination, local fatigue levels, and other changes related to neuromuscular activity (20, 21) found that the average Root Mean Square (RMS) values extracted from sEMG signals of agonist and antagonist muscles could quantitatively assess spasticity in stroke patients. EEG enables the assessment of neural activity and functions of large-scale cortical networks due to its excellent temporal resolution (22). Electroencephalography (EEG) is a widely used neurophysiologic technique that is commonly used for the diagnosis of brain-related disorders and assessment of cognitive function because it is inexpensive and easy to monitor at the bedside (23). EEG is the electrophysiologic technique with the highest temporal resolution, on the order of milliseconds, which can directly measure neural activity in real-time, record oscillations in the electrical activity of neurons under the scalp electrodes, as well as reflect the overall functional state of the cerebral nervous system, and is suitable for the study of brain networks and their functional dynamics (24). There are numerous methods for analyzing EEG, and the more commonly used ones are time-domain analysis, frequency-domain analysis, time-frequency analysis, and spatial-domain analysis. EEG microstate is an emerging concept in the field of neuroscience, and with the continuous development of EEG technology, more and more studies have focused on EEG microstate to reveal their role in brain information processing and consciousness regulation (25, 26). In 1987, Lehmann et al. (27) first introduced the concept of EEG microstate, suggesting that spontaneous EEG activity in the resting state can be described by a limited number of topographies of scalp potentials and that the topography of the topographies always remains relatively stable for 60–120 ms, after which it rapidly transitions to another relatively stable topographic structure.

Koenig et al. (28) found that resting-state EEG signals could be clustered into four classical EEG microstate types, microstate A, microstate B, microstate C, and microstate D, by performing microstate analysis. These four microstates can explain most of the EEG changes in the resting state and have good reliability and stability (29). They are not random patterns but are believed to reflect the synchronized activity of large-scale resting-state networks (RSNs) as identified by functional magnetic resonance imaging (fMRI) (30). Microstate A is associated with the auditory network and phonological processing. Microstate B is linked to the visual network and visual perception. Microstate C is correlated with the salience network and is involved in interoceptive-autonomic processing and cognitive control. Microstate D is related to the attention network, particularly dorsal attention, and is involved in goal-directed cognitive functions. This functional attribution makes microstate analysis a powerful tool for investigating rapid temporal dynamics of large-scale brain networks in cognitive tasks and neurological disorders (30). In the context of motor recovery, alterations in these microstates may provide a unique window into the cortical reorganization process after stroke.

Furthermore, these four classes of microstates are thought to represent the synchronized activity of large-scale network nodes that can correspond to resting-state brain networks found in fMRI. Britz et al. (31) explored the relationship between EEG microstate activity and fMRI activation using a generalized linear model in simultaneous EEG-fMRI recordings and found that four EEG microstates (A, B, C, and D) could correspond to four resting-state functional brain networks: auditory, visual, salience, and attentional networks, respectively. Resting-state EEG microstates have been studied in a variety of neuropsychiatric disorders such as depression (32), obsessive-compulsive disorder (33), and Alzheimer’s disease (34–36), but they have rarely been used in the analysis of movement disorders in stroke. Microstate analysis is a unique EEG analysis method that can capture specific spatio-temporal patterns of brain electrical activity. These microstates reflect the state of the brain in different cognitive and sensory tasks and the neural mechanisms associated with them (29, 37). Wang et al. (38) revealed the shortened duration of microstate C in stroke patients positively correlates with Fugl-Meyer assessment scores, indicating potential associations between reduced neuroplasticity in specific microstates and the extent of motor function recovery. Furthermore, stroke patients differ significantly from the healthy control group in most parameters of microstates A, B, and C (39). This implies distinct alterations in EEG activity across multiple microstates, potentially associated with brain damage and neural network reorganization resulting from stroke. Notably, Rubega et al. (2) observed that left-hemisphere stroke patients exhibited higher global explained variance, occurrence rate, and coverage of microstate D compared to right-hemisphere stroke survivors. This suggests that different stroke types and lesion locations may have varying effects on microstates, leading to disparities between left and right-sided stroke patients. These studies highlight the potential of EEG microstate analysis in exploring the neurophysiological mechanisms of stroke patients and provide crucial insights for a deeper understanding of the impact of stroke. While prior studies have combined rTMS with EEG or EMG (40), few have integrated all three modalities to assess stroke rehabilitation. Recent work by Rubega et al. (2) linked EEG microstates to hemispheric lateralization post-stroke, but neurophysiological correlates of rTMS-induced motor recovery remain unexplored. Our study bridges this gap by synchronously quantifying cortical dynamics (EEG microstates), corticospinal output (EMG), and clinical outcomes.

In the field of neuroscience, the connection between EEG microstates and target muscles has been a subject of much research interest. This connection involves complex nerve conduction processes and muscle control mechanisms that are critical to our understanding of human motor behavior and nervous system function. Electroencephalographic microstates are transient and stable patterns of electrical activity that the brain exhibits during a specific time window. These electrical activity patterns reflect the activity state of neurons in the brain and are the basis of information processing in the brain. The target muscle is the muscle group that carries the main load in a particular task or movement. The selection and synergy of target muscles is key to motor control, and this process is regulated by the nervous system. rTMS provides insights into the neurofunctional changes occurring in the brain, while EMG records muscle activity and microstate analysis reveals detailed patterns of brain electrical activity. Because of the substantial distinctions in structure and function between the two hemispheres of the brain, this study concentrated on collecting data from stroke patients with right-sided hemiplegia to ensure greater consistency in the results. Therefore, this study investigated the effects of repetitive transcranial magnetic stimulation (rTMS) on upper limb motor recovery in patients with right hemiplegia after stroke and healthy controls, integrating the analysis of EEG microstates and EMG to assess neuromuscular changes.

## 2 Materials and methods

### 2.1 Subjects

A convenience sample of 20 stroke patients (13 males and seven females) were recruited from the Seventh Affiliated Hospital of Sun Yat-sen University, with an average age of 63.10 ± 10.70 years old. This sample size is comparable to previous pioneering studies investigating EEG microstates and rTMS in stroke populations (2, 38) and was deemed sufficient for an initial exploration of the proposed neurophysiological biomarkers. All patients were right-handed with right-sided hemiparesis. The characteristics of patients are shown in Table 1. Twenty right-handed subjects were recruited as the healthy control group (HC), consisting of 10 males and 10 females, with an average age of 41.55 ± 17.27.

**Table 1:** Demographic characteristics of stroke patients.

Subject	Sex	Age	Affected side	Stroke type	FMA-UE		ARAT	
					Pre-treatment	Post-treatment	Pre-treatment	Post-treatment
1	M	59	Right	Ischemic	4	7	0	0
2	M	69	Right	Ischemic	47	49	43	47
3	F	80	Right	Ischemic	49	49	46	48
4	M	48	Right	Hemorrhagic	13	13	0	0
5	M	55	Right	Ischemic	60	60	54	54
6	M	66	Right	Ischemic	64	64	54	54
7	F	58	Right	Ischemic	8	9	0	0
8	M	64	Right	Hemorrhagic	63	63	54	54
9	M	48	Right	Hemorrhagic	8	12	0	0
10	F	58	Right	Ischemic	7	11	0	0
11	F	59	Right	Ischemic	13	13	2	2
12	M	42	Right	Hemorrhagic	32	39	26	30
13	M	68	Right	Ischemic	10	12	4	4
14	F	55	Right	Hemorrhagic	7	7	0	0
15	M	75	Right	Ischemic	54	59	54	55
16	M	60	Right	Hemorrhagic	33	38	35	39
17	M	73	Right	Ischemic	52	57	53	55
18	F	80	Right	Ischemic	36	39	36	38
19	F	73	Right	Ischemic	18	19	10	10
20	M	72	Right	Ischemic	52	53	55	55

M, male; F, female.

Inclusion criteria: (1) Cerebral hemorrhage or cerebral infarction confirmed by computerized tomography or magnetic resonance imaging; (2) All showed unilateral limb dysfunction; (3) the first onset of the disease with a duration between 2 weeks and 6 months; (4) Patients aged from 18 to 80 years old with normal vital signs and stable condition; (5) no severe cognitive dysfunction, clear consciousness, can understand and follow simple instructions and tasks to complete the action; (6) Informed consent was signed by the patients or their family members.

Exclusion criteria: (1) dysfunction caused by lesions in the cerebellum or brainstem; (2) Balance dysfunction due to severe orthopedic diseases such as vestibular nerve dysfunction, peripheral neuropathy, or lower extremity musculoskeletal disorders; (3) severe visual field defect, unilateral neglect, and other visual function impairment, hearing or severe cognitive dysfunction cannot understand and complete the movement; (4) complicated with serious heart, lung, liver or kidney diseases, uncontrolled hypertension or malignant tumors that cannot be trained; (5) patients with bilateral lower limb dysfunction, Alzheimer’s disease, Parkinson’s disease, epilepsy, and other nervous system diseases.

Healthy Controls (HC): Age 18–80 years, no neurological or psychiatric history, normal cognitive function, no contraindications for EEG or EMG.

This research obtained ethical approval from the Committee on Medical Ethics at the Seventh Affiliated Hospital of Sun Yat-sen University Ethical (NO. KY-2023-090-2), ethical approval was granted on November 2, 2023 and all procedures adhered to the principles outlined in the Declaration of Helsinki. Details of trial protocol registration can be seen in chictr.org.cn (chictr.org.cn Identifier: ChiCTR2100049509).

### 2.2 rTMS treatment

**Figure 1:**
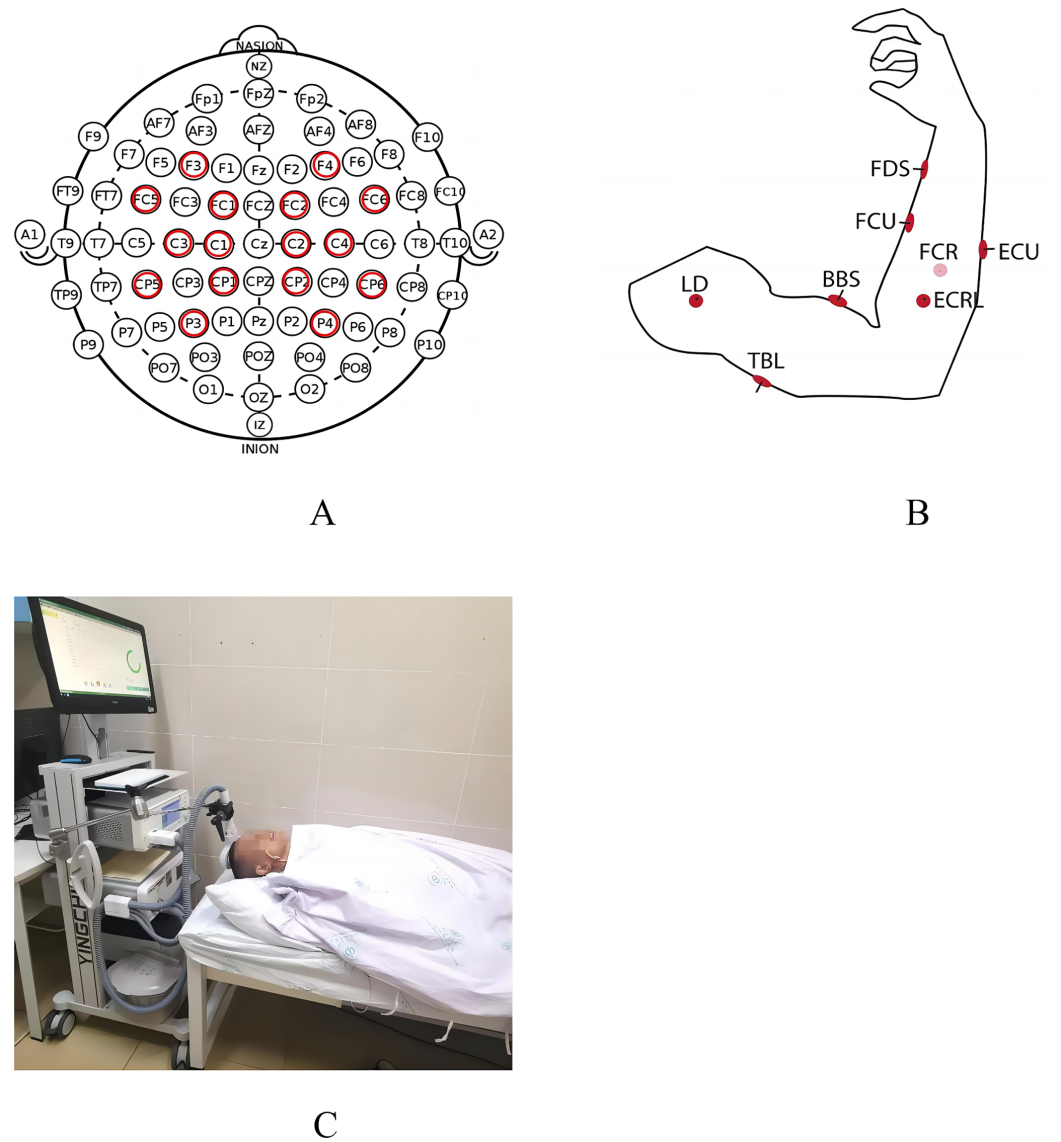
Channel acquisition schematic and treatment schematic. **(A)** The location of the EEG channel. **(B)** The location of the EMG channel. **(C)** rTMS in stroke patients.

The rTMS device (M-100 Ultimate model, Shenzhen Yingzhi Technology Co., Ltd., China) equipped with a ‘Figure-8’ coil (each side with a diameter of 8.5 cm) was used to treat patients, as shown in Figure 1C. The primary motor cortex (M1) is the area of the cerebral cortex primarily responsible for controlling limb movements. By stimulating the M1 area, the excitability of motor neurons can be directly affected, thus improving motor function. An International 10–20 EEG system was used for localization. The target stimulation site was the primary motor cortex (M1) of the affected hemisphere, corresponding to the C3 electrode position for right-hemiplegic patients. Before treatment, assessing the patient’s resting motor threshold was imperative. Patients were positioned supine on a treatment bed, and the Figure-8 coil was placed over M1. The coil was placed tangentially to the skull surface with the handle oriented at 45° to the midsagittal line. Single-pulse TMS was delivered while visually monitoring the flexor digitorum superficialis of the contralateral limb for muscle twitches. The resting motor threshold (RMT) was defined as the minimum intensity required to elicit a visible contraction of the middle finger flexor muscle in ≥5/10 consecutive trials.

After determining the RMT, patients were instructed to lie supine on a treatment bed, maintain a state of wakefulness, and relax their entire body, avoiding any head movements throughout the procedure. The ‘Figure-8’ coil was positioned over the targeted stimulation site, ensuring stable coil placement, and the site was stimulated accordingly. The treatment parameters set for this study were as follows: Stimulation frequency of 10 Hz, stimulation intensity set at 100% of RMT, 2-s stimulation duration, 8-s interstimulus interval, repeated for a total of 60 sequences, resulting in 1200 pulses. The treatment duration was 10 min, and the target stimulation site was the affected hemisphere’s M1 area. Patients received rTMS treatment once daily for a total of 5 sessions. Clinical outcome measures were assessed at baseline and at the end of all treatments by an experienced rehabilitation therapist who was familiar with the scales and tests used in this study. Clinical outcome measures included the Upper Extremity Section of the Fugl-Meyer Assessment (FMA-UE) and the Action Research Arm Test (ARAT), in which higher scores indicate better upper extremity function. Resting-state EEG and EMG data were collected from healthy subjects; resting-state EEG and EMG data were collected from stroke subjects before the start of treatment and after the completion of treatment.

### 2.3 Synchronized EEG and EMG acquisition

The study focused on the brain activity of the motor cortex. A wireless 16-channel EEG system (BLueBrain. ZT32, Xi’an Blue Brain Co. Ltd., China) was used to collect signals from the motor cortex according to the international 10–20 system. The EEG electrodes in the motor cortex are shown in Figure 1A, consisting of 16 channels (P4, CP2, FC5, C3, P3, C2, FC6, C4, CP6, F3, FC2, FC1, F4, CP5, C1, CP1). The EEG acquisition system applied a 0.1–100 Hz band-pass filter to the signal digitized with a sampling rate of 1,000 Hz. All electrode impedances were maintained below 10 kΩ. The resting-state EEG data with eyes closed were collected.

Muscle activity was recorded at 1000 Hz with surface electrodes for an EMG amplifier (BLueBCI-8, Xi’an Blue Brain Co. Ltd., China). The reference electrode was placed at the wrist ligaments. Pairs of electrodes were placed on the following 10 muscles of the affected upper extremity for patients or the right arm for healthy participants, as shown in Figure 1B: (1) flexor digitorum superficialis (FDS), (2) flexor carpi ulnaris (FCU), (3) flexor carpi radiali (FCR), (4) extensor carpi ulnaris (ECU), (5) extensor carpi radialis long (ECRL), (6) biceps brachii (BB), (7) triceps brachii (TB), (8) deltoid medius (DM), (9) deltoid anterior (DA), (10) deltoid posterior (DP).

### 2.4 Pre-processing of EEG and EMG

We employed the EEGLab application for the analysis and processing of EEG and EMG signals. A 0.5–45 Hz band-pass filter and a 50 Hz notch filter were applied to the EEG data using a finite impulse response filter. EMG data were band-passed between 20 and 400 Hz. The continuous EEG and EMG were downsampled to 500 Hz. The EEG data remontage to a common average reference. Hereafter, the EEG and EMG time series were epoched into segments of 2 s duration. These segments were then visually inspected to remove artifacts (i.e., eye movements, cardiac activity, and scalp muscle contraction) using the independent component analysis procedure to identify and extract visual artifact components. Finally, any EEG epochs with amplitude values exceeding ± 80 μV at the electrodes were rejected.

### 2.5 Microstate analysis of the motor cortex

Microstate analysis followed standardized methods (41) using CARTOOL software (42). The multichannel EEG signal is considered a series of instantaneous topographies of electric potentials. The peak position of the global field power (GFP) curve represents the moment of the strongest field intensity and the highest topographic signal-to-noise ratio (43).

In the first step, based on the potential values of individual electrodes for each subject, the GFP of the EEG signal at a specific time is calculated, as defined in Equation 1:


(1) $$\mathrm{GFP}(t)=\sqrt{\frac{\sum_{i=1}^{n}\;{\left(v_{i}(t)-\bar{v}(t)\right)}^{2}}{n}}$$

where *v_i_(t)* is the voltage at electrode *I* at time *t*, $\bar{v}(t)$ is the mean voltage across all electrodes at time *t*, and *n* is the number of electrodes.

Next, the GFP peak time series is extracted. Since the EEG topographical maps at the times of GFP peaks have the highest signal-to-noise ratio, the maps between the two peaks tend to exhibit similar structures. The K-means clustering algorithm is used on the GFP peak time series in the second step. Five EEG topographical maps are randomly selected as initial cluster centers. The remaining maps are compared to the initial centers, and each map is labeled with the cluster center that it is most related to. New cluster centers are computed, and this process is repeated until no further improvement is achieved. The resulting five cluster centers constitute the microstate model. In the third step, the microstate model is used to calculate intra-group ‘microstate models’ for all subjects, thereby categorizing the topographical maps into five classes labeled as microstates A, B, C, D, and E. These microstates exhibit spatial configurations in the right-frontal, left-posterior, left-frontal, midline frontal, and central parietal directions, respectively.

In the fourth step, competitive fitting uses spatial correlation to associate the original EEG data with the five microstate models. Each time point of the original EEG data is labeled with the highest correlation microstate, resulting in a temporal evolution of EEG topographical maps. Further, microstate-specific parameters are extracted for analysis. Microstate time series contain rich neurophysiological information. In microstate analysis, key parameters include mean duration, temporal coverage, and frequency of occurrence, which provide important information about changes in brain states:

(1) Average duration: refers to the average length of time that each microstate appears and remains stable. This parameter reflects the stability of subcortical neuronal activity.
(2) Temporal coverage: indicates the percentage of the total recording time during which such microstates were dominant. This parameter represents the relative percentage of time coverage relative to other neurogenic patterns.
(3) Frequency of occurrence: indicates the average number of times per second that the microstate was dominant during the recording period. This parameter reflects the tendency or probability that a potential neuron or nervous system is activated.

### 2.6 EMG analysis

The analysis of EMG signals mainly includes the analysis of raw surface EMG signals and the analysis of processed data, and the data analysis mainly focuses on both time domain and frequency domain analysis. Here we extract the peak-to-peak and root-mean-square values from the EMG signal:

(1) Peak-to-peak (PTP) has been used to assess the force of a muscle contraction as the difference between the maximum and minimum amplitude values in the EMG signal. It provides information on the amplitude range of the signal, which can be used to detect the maximum activation level of the muscle and the amplitude change of the signal.

PTP=max(x)−min(x)

where *x* represents the sample value of the EMG signal.

(2) The root mean square (RMS) values provide information about signal strength and activation levels and are commonly used to analyze the strength of electrical activity in muscles. It is calculated by squaring the amplitude of the individual data points of the signal, taking the average, and then taking the square root.

$$\mathrm{RMS}=\sqrt{\frac{1}{N}\sum_{i=1}^{N}\;x_{i}^{2}}$$

where $x_{i}\;$represents the *i* sample value of the EMG signal and $\bar{N}\;$is the total number of sample points.

### 2.7 Statistical analysis

All the statistical analyses were performed using SPSS (23.0; SPSS, Inc., Chicago, IL, United States). The Wilcoxon test was used for non-normally distributed data. Two-way analyses of variance (ANOVA) with subject factor for the group (patient group and health control group) and microstates (A/B/C/D/E) were performed for microstate duration, time coverage, and insurance. Two-factor remeasurement ANOVA was applied to detect treatment effects. The level of significance was set at *p* < 0.05. The Bonferroni-corrected method was performed for *post hoc* testing of significant main effects to minimize the risk of type I error, whereas simple effect analysis was used for testing significant interactions. The Pearson correlation analysis was used to detect correlations between microstates and EMG indicators before and after treatment.

## 3 Results

### 3.1 Clinical outcomes

The changes in clinical outcomes before and after treatment in the stroke group were calculated as shown in Table 2. After 5 days of rTMS intervention, the FMA-UE scores and ARAT scores of the stroke test group were significantly improved compared to the pre-treatment period, and the difference was statistically significant (*p* < 0.05).

**Table 2:** Clinical outcomes of stroke patients.

Outcomes	Pre-treatment	Post-treatment	*t*	*p*
FMA-UE	31.50 ± 21.97	33.65 ± 21.92	4.357	<0.001***
ARAT	26.30 ± 24.00	27.25 ± 24.57	2.826	0.011*

FMA-UE, the Upper Extremity Section of the Fugl-Meyer Assessment; ARAT, the Action Research Arm Test.

*p < 0.05, ***p < 0.001.

### 3.2 EMG results

**Figure 2:**
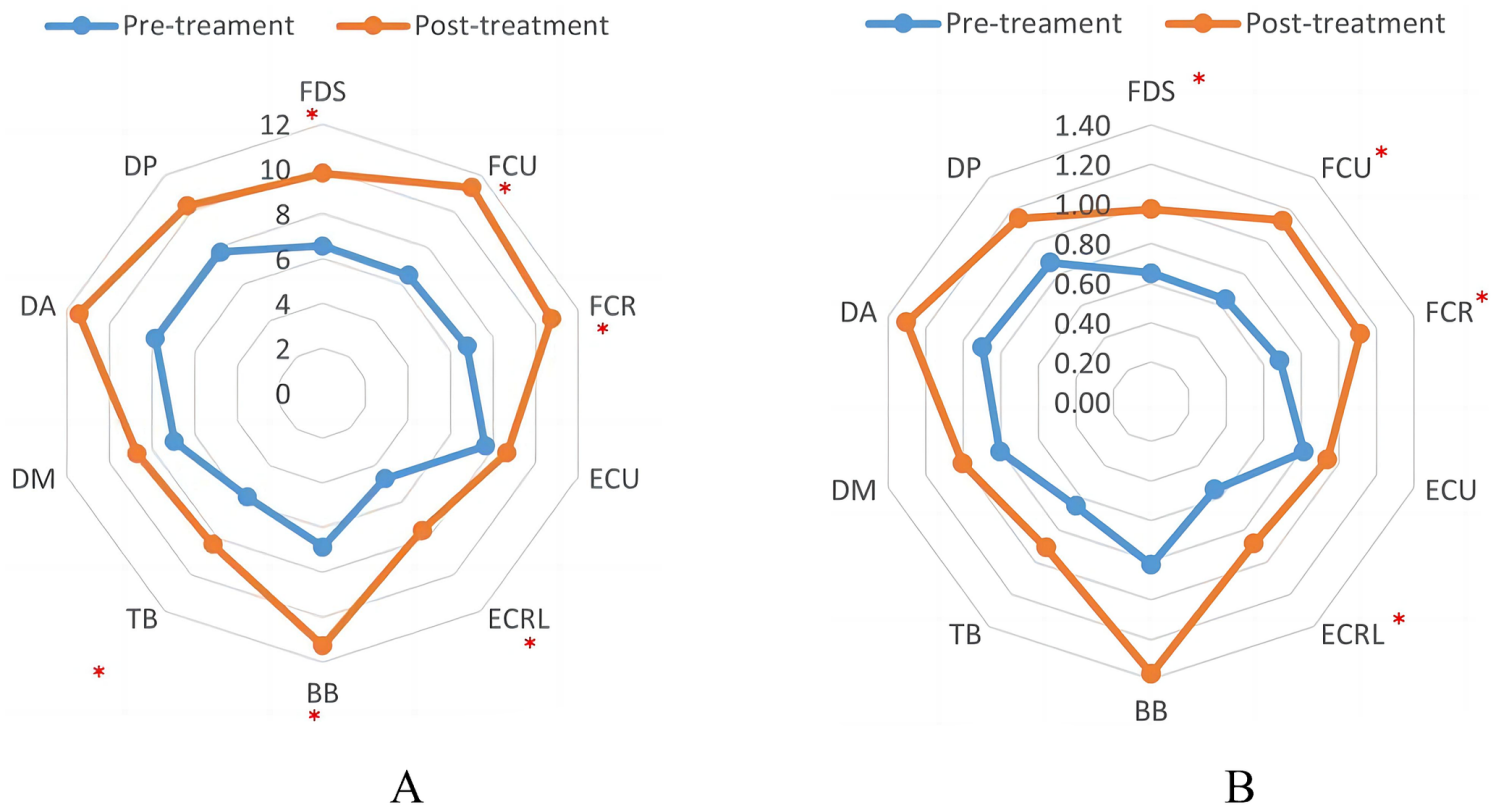
Indicator of EMS in patients before and after TMS treatment. **(A)** The peak-to-peak value of EMG. **(B)** The root-mean-square value of EMG. ∗*p* < 0.05.

The mean PTP and RMS values of EMG were calculated as shown in Table 3. Initially, EMG results were compared between the HC and patient groups. The results revealed that the PTP and RMS values for all 10 muscles were lower in the patient group than in the HC group. Significant differences were observed among the extensor carpi ulnaris, extensor carpi radialis longus, and triceps brachii (*p* < 0.05). These results indicate a reduction in muscle contraction capability among the patients. EMG values before and after rTMS treatment are shown in Figure 2. After rTMS treatment, there was a significant increase in both the RMS and PTP values for muscle groups flexor digitorum superficial, flexor carpi ulnaris, flexor carpi radial, extensor carpi ulnaris, extensor carpi radialis longus, biceps brachi, and triceps brachii (*p* < 0.05).

**Table 3:** Peak-to-peak and RMS values of EMG.

Muscle	Peak to Peak			RMS		
	HC	Pre	Post	HC	Pre	Post
FDS	8.73 ± 7.08	6.56 ± 5.62	9.81 ± 7.15	0.96 ± 0.74	0.65 ± 0.42	0.97 ± 0.54
FCU	8.92 ± 7.01	6.50 ± 4.10	11.33 ± 8.25	0.97 ± 0.72	0.64 ± 0.26	1.13 ± 0.66
FCR	9.42 ± 6.74	6.78 ± 6.00	10.74 ± 6.31	1.01 ± 0.70	0.68 ± 0.51	1.11 ± 0.54
ECU	14.84 ± 8.83	7.62 ± 5.67*	8.64 ± 5.38	1.51 ± 0.90	0.81 ± 0.55	0.94 ± 0.52
ECRL	10.64 ± 7.67	4.73 ± 1.30**	7.55 ± 3.73	1.13 ± 0.77	0.55 ± 0.13	0.89 ± 0.44
BB	6.94 ± 3.67	6.86 ± 3.19	11.26 ± 8.06	0.80 ± 0.40	0.82 ± 0.42	1.37 ± 1.03
TB	14.91 ± 10.10	5.72 ± 4.48**	8.34 ± 4.43	1.65 ± 1.08	0.65 ± 0.48	0.91 ± 0.41
DM	6.77 ± 5.10	6.96 ± 9.23	8.71 ± 4.98	0.82 ± 0.60	0.81 ± 1.04	1.00 ± 0.54
DA	9.85 ± 11.48	7.87 ± 8.04	11.46 ± 13.76	1.23 ± 1.45	0.90 ± 0.98	1.30 ± 1.52
DP	8.44 ± 6.24	7.77 ± 7.82	10.31 ± 6.66	1.01 ± 0.74	0.87 ± 0.85	1.14 ± 0.61

FDS, flexor digitorum superficialis; FCU, flexor carpi ulnaris; FCR, flexor carpi radiali; ECU, extensor carpi ulnaris; ECRL, extensor carpi radialis long; BB, biceps brachii; TB, triceps brachii; DM, deltoid medius; DA, deltoid anterior; DP, deltoid posterior. *p < 0.05, **p < 0.01.

### 3.3 Microstate characteristics of the motor cortex in stroke patients

**Figure 3:**
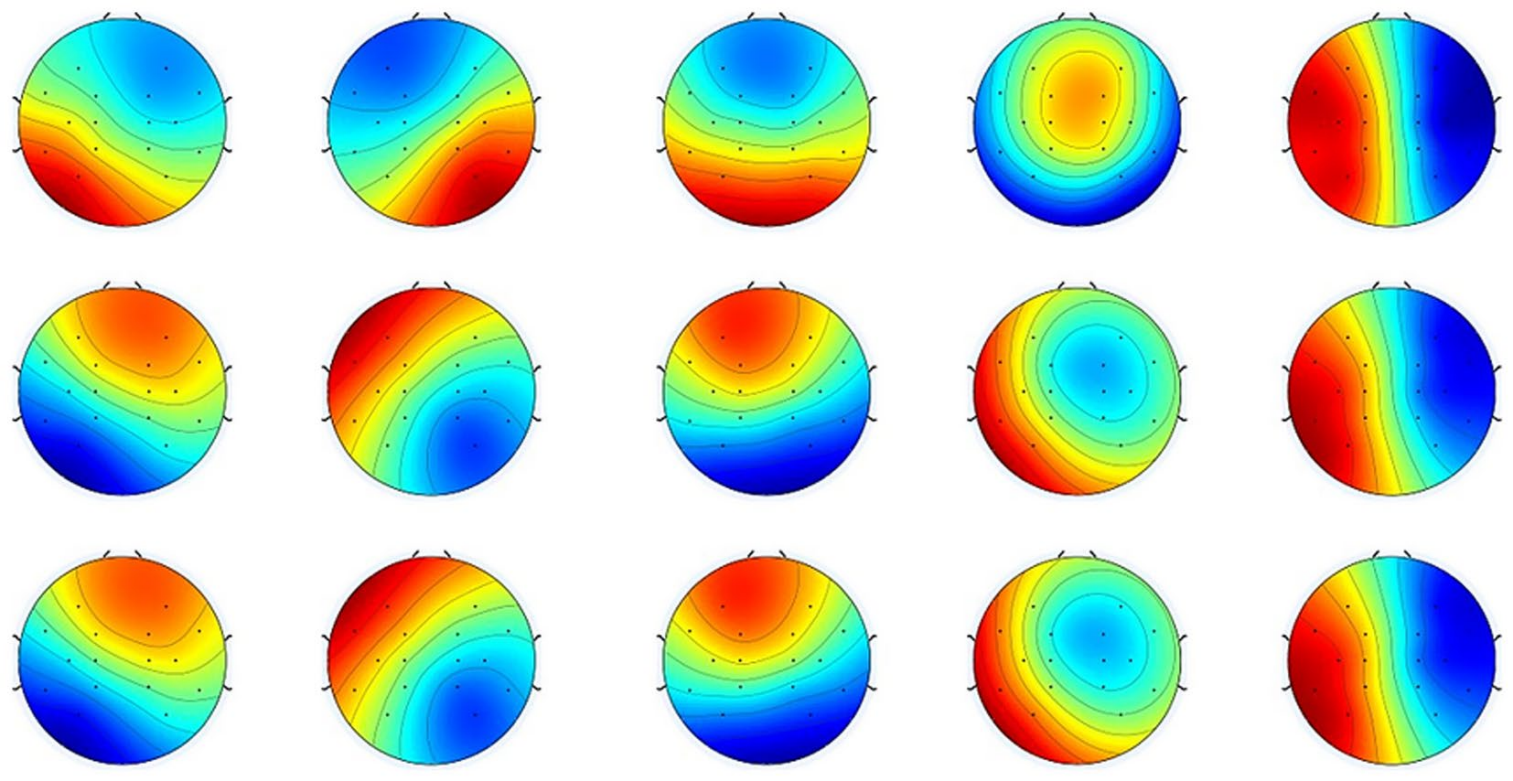
Mean normalized topographical maps of microstate classes A, B, C, D, and E. The HC and patient groups have similar microstate classifications of resting-state EEG. Red and blue indicate positive and negative values, respectively. The polarity is ignored during microstate analysis (22, 29, 64).

**Figure 4:**
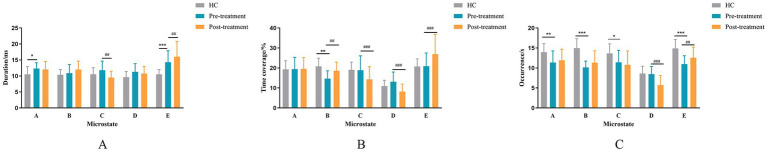
Mean and standard error of the HC and patient groups. **(A)** The mean duration for microstate classes (A–E). **(B)** The time coverage for microstate classes (A–E). **(C)** The occurrence rate for microstate classes (A–E). * indicate a significant difference between the HC and pre-treatment in patient groups as assessed by the independent samples *t*-test after false discovery rate correction. *p < 0.05, **p < 0 0.01, ***p < 0 0.001. The # indicates a significant difference between the pre-treatment and post-treatment in patient groups as assessed by the pared-samples *t*-test after false discovery rate correction. ^#^*p* < 0.05, ^##^*p* < 0 0.01, ^###^*p* < 0.001.

The cluster number of optimal microstates for the patients and HC groups is five, as shown in Figure 3. The mean and standard error of the HC and patient groups were analyzed as shown in Figure 4. Duration, time coverage, and occurrences of microstates were analyzed as shown in Table 4. Compared to healthy controls, stroke patients at baseline exhibited distinct microstate abnormalities in Figure 4. Most notably, the duration of microstates A and D was significantly prolonged in patients (*p* < 0.05, Figure 4A), while the occurrence of microstate C was significantly reduced (*p* < 0.001, Figure 4C). These findings suggest a stroke-induced alteration in the temporal dynamics of brain networks associated with these microstates. The results also revealed a significant interaction effect between group and microstate class for time coverage, *F*(4, 144) = 10.05, *p* = 0.000, *η^2^* = 0.30. Further *post hoc* analyses found that, in microstate A, time coverage was significantly higher in the patient group than in the HC group, *p* = 0.033 (Figure 4B). The result revealed a significant interaction effect between group and microstate class for occurrences *F*(4, 144) = 14.68, *p* = 0.000, *η^2^* = 0.39. A simple effects analysis found a significant main effect of group on microstate C, *F*(11, 48) = 16.54, *p* < 0.000. Further *post hoc* analyses revealed that the occurrence of microstate C in the patient group was significantly lower than in the HC group (Figure 4C).

**Table 4:** The temporal properties of HC, pre-treatment, and post-treatment groups.

Microstate parameter	HC	Pre	Post	HC vs. Pre [*p* (t)]	Pre vs. Post [*p* (t)]
Duration					
Class A	10.50 ± 2.42	12.33 ± 1.81	12.06 ± 2.49	0.019 (−2.48)	0.616 (0.509)
Class B	10.39 ± 1.63	10.88 ± 2.69	12.01 ± 2.64	0.556 (−0.59)	0.05 (−2.08)
Class C	10.54 ± 2.03	11.80 ± 2.85	9.51 ± 1.99	0.179 (−1.38)	0.001 (4.15)
Class D	9.64 ± 1.76	11.30 ± 2.54	10.77 ± 2.21	0.050 (−2.08)	0.180 (1.39)
Class E	10.51 ± 1.49	14.30 ± 3.59	16.01 ± 4.81	0.001 (−3.60)	0.007 (−3.03)
Time coverage					
Class A	19.32 ± 4.29	19.42 ± 5.92	19.57 ± 5.64	0.956 (−0.06)	0.911 (−0.113)
Class B	20.81 ± 4.11	14.67 ± 3.92	18.60 ± 4.36	0.001 (3.52)	0.001 (−3.78)
Class C	19.09 ± 3.83	18.88 ± 7.20	14.33 ± 6.31	0.923 (0.10)	0.009 (2.927)
Class D	10.96 ± 2.92	13.14 ± 4.85	8.21 ± 3.78	0.156 (−1.46)	0.000 (5.355)
Class E	20.75 ± 3.81	20.88 ± 6.62	26.94 ± 9.88	0.952 (−0.06)	0.000 (−4.260)
Occurrence					
Class A	13.95 ± 2.13	11.35 ± 2.90	11.93 ± 2.77	0.009 (2.78)	0.311 (−1.04)
Class B	14.95 ± 2.36	10.14 ± 1.59	11.31 ± 2.98	0.000 (7.01)	0.059 (−2.01)
Class C	13.64 ± 2.38	11.41 ± 2.99	10.78 ± 3.47	0.031 (2.27)	0.361 (0.94)
Class D	8.62 ± 1.82	8.48 ± 1.89	5.73 ± 2.36	0.833 (0.21)	0.000 (5.91)
Class E	14.89 ± 2.24	10.95 ± 2.10	12.57 ± 2.64	0.000 (5.13)	0.002 (−3.66)

### 3.4 Microstates change of rTMS treatment in stroke patients

The duration, time coverage, and occurrence of microstates before and after rTMS treatment were analyzed. The results are shown in Table 4. The mean and standard error of the HC and patient groups were analyzed as shown in Figure 4. The interaction effect of treatment times and microstates for microstate duration was significant, *F*(4, 120) = 34.09, *p* < 0.001, *η^2^* = 0.53. Further post-hoc analyses revealed that the durations of microstate E were significantly increased (*p* = 0.007), whereas the duration of microstate C was significantly reduced (*p* = 0.001) in the post-treatment compared to the pre-treatment (Figure 4A). The interaction effect of treatment times and microstates for the time coverage of microstate was significant *F*(4, 76) = 13.51, *p* < 0.001, *η^2^* = 0.42. Further *post hoc* analyses revealed that the time coverage of microstates B and E was significantly increased post-treatment than pre-treatment; the time coverage of microstates C and D was significantly reduced post-treatment than pre-treatment (Figure 4B). The interaction effect of treatment times and microstates for the occurrence of microstate was significant *F*(4, 76) = 24.38, *p* < 0.001, *η^2^* = 0.44. Further *post hoc* analyses revealed a significant decrease in the occurrence of microstate D and a significant increase in the occurrence of microstate E in the post-treatment compared to the pre-treatment period (Figure 4C).

### 3.5 Relationship between muscle recovery and microstate of motor cortex

The results of the correlation analysis between the EEG microstate and the PTP values of EMG indexes before and after treatment in the stroke test group are shown in Table 5. The time coverage of microstate A was positively correlated with the PTP enhancement in the biceps brachii (*r* = 0.532, *p* = 0.041). The occurrence of microstate A was positively correlated with PTP enhancement in the radial long wrist extensor (*r* = 0.515, *p* = 0.049) and biceps brachii (*r* = 0.673, *p* = 0.006). The frequency of microstate B was positively correlated with PTP enhancement in biceps brachii (*r* = 0.594, *p* = 0.019). The time coverage of microstate C was positively correlated with PTP enhancement in biceps brachii (*r* = 0.562, *p* = 0.029). The occurrence of microstate C was positively correlated with PTP enhancement in biceps brachii (*r* = 0.615, *p* = 0.015).

**Table 5:** Correlation analysis of microstate parameters and EMG PTP values before and after treatment in patient group.

Microstate parameter	FDS	FCU	FCR	ECU	ECRL	BB	TB	DM	DA	DP
Duration										
Class A	−0.154	−0.064	0.029	−0.190	0.017	0.037	0.160	0.286	0.267	0.039
Class B	−0.112	−0.054	0.206	0.152	−0.099	−0.177	0.003	−0.051	0.171	−0.231
Class C	−0.187	−0.137	−0.358	−0.322	0.086	0.185	−0.043	0.227	−0.087	−0.012
Class D	0.040	0.117	0.090	−0.129	−0.062	−0.161	−0.061	−0.149	−0.469	−0.023
Class E	−0.201	−0.318	0.099	−0.184	0.067	−0.434	−0.269	−0.464	−0.012	−0.479
Time coverage										
Class A	0.185	0.206	0.252	0.220	0.416	0.532*	0.171	0.262	0.219	0.277
Class B	0.142	0.110	0.135	0.354	0.070	0.379	0.088	0.177	0.161	0.133
Class C	0.130	0.083	−0.091	0.109	0.351	0.562*	0.073	0.259	0.063	0.217
Class D	0.271	0.281	0.231	0.261	0.205	0.213	0.020	−0.064	−0.277	0.158
Class E	0.002	−0.160	0.197	0.077	0.296	−0.042	−0.204	−0.362	0.071	−0.075
Occurrence										
Class A	0.408	0.355	0.312	0.404	0.515*	0.673**	0.148	0.165	0.083	0.293
Class B	0.307	0.244	0.066	0.248	0.166	0.594*	0.098	0.239	0.031	0.280
Class C	0.290	0.204	0.093	0.282	0.424	0.615*	0.090	0.183	0.048	0.274
Class D	0.407	0.403	0.433	0.508	0.416	0.427	0.057	−0.042	−0.080	0.264
Class E	0.225	0.122	0.155	0.092	0.331	0.323	−0.086	−0.105	−0.169	0.286

FDS, flexor digitorum superficialis; FCU, flexor carpi ulnaris; FCR, flexor carpi radiali; ECU, extensor carpi ulnaris; ECRL, extensor carpi radialis long; BB, biceps brachii; TB, triceps brachii; DM, deltoid medius; DA, deltoid anterior; DP, deltoid posterior. *p < 0.05, ***p < 0.001.

The correlation analysis results of EEG microstate and EMG index RMS values before and after treatment in the patient group are shown in Table 6. The results showed that the duration of microstate A was negatively correlated with the RMS enhancement of flexor digitorum superficialis (*r* = −0.727, *p* = 0.002) and biceps brachii (*r* = −0.519, *p* = 0.047). The time coverage of microstate A was negatively correlated with the RMS enhancement of biceps brachii (*r* = −0.548, *p* = 0.034). The occurrence of microstate A was positively correlated with the RMS enhancement of triceps brachii (*r* = 0.528, *p* = 0.043). The duration of microstate B was negatively correlated with the RMS enhancement of flexor digitorum superficialis (*r* = −0.542, *p* = 0.037). The time coverage of microstate C was positively correlated with the RMS enhancement of the middle deltoid tract (*r* = 0.594, *p* = 0.045). The occurrence of microstate D was positively correlated with the RMS enhancement of flexor digitorum superficialis (*r* = 0.683, *p* = 0.005) and extensor carpi radialis longus (*r* = 0.557, *p* = 0.031). The occurrence of microstate E was positively correlated with the RMS enhancement of flexor digitorum superficialis.

**Table 6:** Correlation analysis of microstate parameters and myoelectric RMS values before and after treatment in patient group.

Microstate Parameter	FDS	FCU	FCR	ECU	ECRL	BB	TB	DM	DA	DP
Duration										
Class A	−0.727**	−0.178	−0.114	0.081	−0.273	−0.519*	0.046	0.026	0.440	0.206
Class B	−0.542*	−0.224	−0.019	0.185	0.080	0.084	−0.360	0.266	−0.037	0.208
Class C	−0.229	−0.155	−0.267	−0.374	−0.260	−0.257	0.209	−0.442	0.207	−0.011
Class D	0.064	0.026	0.101	−0.088	−0.073	0.214	−0.162	0.000	−0.407	−0.325
Class E	−0.440	−0.233	−0.280	0.142	−0.375	0.059	−0.294	0.028	−0.248	−0.176
Time coverage										
Class A	0.043	0.202	0.152	0.304	0.207	−0.548*	0.435	−0.299	0.233	0.099
Class B	0.140	0.091	0.110	0.140	0.424	−0.268	0.020	−0.144	0.040	0.199
Class C	0.263	0.179	−0.012	−0.076	0.192	−0.384	0.462	−0.523*	0.145	0.053
Class D	0.499	0.269	0.273	0.093	0.348	0.171	0.095	−0.137	−0.409	−0.195
Class E	0.257	0.019	−0.118	0.289	−0.053	−0.136	0.015	−0.194	−0.229	−0.194
Occurrence										
Class A	0.473	0.437	0.315	0.317	0.455	−0.295	0.528*	−0.357	−0.014	0.013
Class B	0.456	0.319	0.212	0.076	0.375	−0.351	0.328	−0.335	0.073	0.062
Class C	0.489	0.333	0.144	0.100	0.364	−0.324	0.467	−0.469	0.014	0.010
Class D	0.683**	0.420	0.410	0.364	0.557*	0.051	0.305	−0.198	−0.336	−0.115
Class E	0.767**	0.285	0.115	0.187	0.129	−0.254	0.276	−0.400	−0.182	−0.317

FDS, flexor digitorum superficialis; FCU, flexor carpi ulnaris; FCR, flexor carpi radiali; ECU, extensor carpi ulnaris; ECRL, extensor carpi radialis long; BB, biceps brachii; TB, triceps brachii; DM, deltoid medius; DA, deltoid anterior; DP, deltoid posterior. *p < 0.05, **p < 0.01.

## 4 Discussion

Stroke patients are left with varying degrees of motor dysfunction, the most common of which is upper limb motor dysfunction. The upper limb function is complex and difficult to recover. Currently, treatment methods to restore upper limb function mainly include drug therapy, physical therapy, occupational therapy, and other conventional therapies, but their efficacy is limited (44). It is generally believed that neuroplasticity is the theoretical basis for motor function recovery. As a non-invasive neuromodulation technique, rTMS has been widely used to treat upper limb motor dysfunction after stroke (45). rTMS stimulation can change the cortical excitability of the cerebral hemisphere, reshape the balance between the cerebral hemispheres, transmit to other regions through the neural circuit communicating with the stimulating area in the brain synaptic structure, excite more horizontal neurons, achieve regional reconstruction of cortical function, and promote the functional recovery of stroke patients.

The FMA-UE scale has a good effect in evaluating the motor function rehabilitation of stroke patients, and it can evaluate the motor function of all parts of the limbs. The ARAT scale is mainly used to evaluate the functional status of the upper limbs of post-stroke patients, and most of its test items target the distal motor function of the upper limbs, which can comprehensively reflect the rehabilitation of the upper limbs and the hand function of stroke patients, and it is widely used in the field of stroke rehabilitation research. The results of our study showed that after rTMS treatment, stroke patients showed significant improvement in FMA-UE scale scores and ARAT scale scores compared with the pre-treatment scores. This confirms that high-frequency rTMS can effectively improve upper extremity motor function after stroke, which is consistent with the results of previous studies (46). A large number of studies have shown that applying high-frequency rTMS to the M1 area on the affected side can effectively elevate the cortical nerve excitability on the affected side and improve the motor function of the upper limb on the damaged side (47). Khedr and Fetoh (48)applied high-frequency rTMS stimulation on the affected side to observe the improvement of the function of the upper limb and found that the improvement of the rTMS stimulation group in terms of the improvement of the grip strength as well as the increase of joint mobility was more obvious than that of the sham stimulation group, suggesting that rTMS treatment can improve upper limb motor function after stroke.

Our initial analysis demonstrated a consistent reduction in both PTP and RMS values across all 10 muscles in the patient group compared to healthy controls. This reduction indicates compromised muscle contraction capability in the patient cohort, aligning with the anticipated effects of neuromuscular dysfunction. This finding is consistent with previous research results and supports the notion that stroke patients exhibit significant impairments in muscle function (49, 50). Following transcranial magnetic stimulation (TMS) treatment, a significant increase in both RMS and PTP values was observed for multiple muscle groups, specifically in flexor digitorum superficial, flexor carpi ulnaris, flexor carpi radialis, extensor carpi ulnaris, extensor carpi radialis longus, biceps brachii, and triceps brachii. This observed increase post-rTMS suggests the potential benefits of rTMS therapy for stroke rehabilitation, indicating a positive impact on the recovery of muscle function. Consistent with existing research findings (51, 52), this supports the effectiveness of rTMS as a neurorehabilitation tool, emphasizing its role in enhancing muscle activity in stroke patients. rTMS is known to modulate cortical excitability by inducing electrical currents in the brain. This modulation influences the firing patterns of neurons in the motor cortex, subsequently affecting the activation of motor units in the targeted muscles. The observed increase in RMS and PTP values post-rTMS may reflect enhanced motor unit recruitment and synchronization, leading to more forceful and coordinated muscle contractions. Additionally, rTMS-induced neural plasticity triggers structural and functional changes in synaptic connections within the motor cortex. This could enhance the efficiency of signal transmission from the central nervous system to the muscles, contributing to the observed increase in electromyographic parameters. Enhanced synaptic efficacy may result in more robust and coordinated muscle contractions, providing a neurophysiological basis for the observed improvements in muscle function post-rTMS treatment.

Resting-state neuronal discharges have been recorded in early electrophysiological studies, and more recent studies have shown that resting-state activity occurs coherently in populations of neurons and that entire brain networks remain continuously active at rest (53, 54). One theory suggests that EEG microstates represent discrete mental processes that combine to produce conscious mental activity, i.e., spontaneous mental activity. Thus, microstates are also considered to be “atoms of thought” (27). The networks that are activated during particular microstates represent different states of conscious thought, and each microstate is associated with a different category of thought that collectively constitutes a conscious state. EEG microstates represent relatively slow and highly synchronized patterns of global oscillations that modulate interactions between local brain structures. Several studies have shown a link between EEG microstates and resting-state networks recognized by fMRI, suggesting that the resting-state network for fMRI may be the same as the one that generates the microstates (41, 55). Britz et al. (31) found that microstates A, B, C, and D corresponded to previously identified resting-state networks, namely the auditory network, the visual network, the salience network, and the attention network, respectively. Thus an increasing number of clinical and cognitive neuroscience studies have adopted large-scale EEG microstate methods to assess the electrical activity of large-scale cortical networks.

The EEG microstate analysis serves as a neurophysiological method for investigating and evaluating the brain’s overall functional state in healthy individuals and those with neurological conditions. Observing a significant increase in the duration of microstate A and a significant decrease in its occurrence in stroke patients raises intriguing questions. Shorter durations of microstates suggest premature termination of certain cognitive processes, while the increased occurrence of specific microstates implies a more frequent repetition of fundamental cognitive steps required for executing particular psychological processes (56). This finding may reflect the negative impact of stroke on brain activity patterns, particularly the disruption of functional connections associated with microstate A (57). Stroke typically results in brain damage and functional impairments, including deficits in motor and cognitive functions (58). The increased duration of microstate A may indicate a compensatory mechanism the brain employs to cope with these functional deficits in stroke patients. This compensation mechanism may help stroke patients maintain relatively normal levels of function in certain tasks and activities. Many studies believe that the left temporal lobe and left insular lobe are the main production regions of microstate A, and the medial prefrontal cortex and left occipital lobe mainly affect the spatial configuration of microstate A (59). Various disorders have been associated with abnormalities in the functional network of the temporal lobe, such as depression, mild cognitive impairment, and Alzheimer’s disease (60, 61). However, it is surprising that there was no significant change in microstate A after rTMS treatment. The absence of microstate A changes post-rTMS may reflect suboptimal coil targeting. Computational modeling shows microstate-specific networks require precise electric field orientation (30). Future studies should incorporate neuronavigation to optimize coil placement. This may suggest that microstate A is not sensitive to rTMS intervention or may require more treatment time to observe changes. It may also be related to reduced neuroplasticity in stroke patients, requiring a longer time for changes in microstate A to become evident. This result underscores the complexity of stroke rehabilitation research and suggests that neurorehabilitation strategies may require more personalization and time to achieve optimal results. In addition, we found that the time coverage and occurrence of microstate A were positively correlated with PTP enhancement in upper limb muscles. The changes in microstate A may represent an adaptive mechanism employed by the brain of stroke patients to cope with functional deficits. However, the association of microstate A with rTMS treatment needs further research for clarification. These findings provide valuable clues for understanding stroke rehabilitation and neuroplasticity, but additional work is needed to elucidate the physiological and neural mechanisms behind these observations.

The reduction in time coverage and occurrence frequency of microstate B observed in stroke patients indicates the disruption in brain functional connectivity resulting from stroke, consistent with previous studies (57, 62). However, following rTMS treatment, a significant increase in the time coverage of microstate B was noted, approaching levels seen in the healthy control group. This finding implies a potentially positive role of rTMS in neurorehabilitation for stroke patients, particularly in terms of rehabilitation goals tied to microstate B. Notably, the findings showed that the frequency of microstate B was positively correlated with PTP enhancement in upper limb muscles. This observation underscores the intimate connection between microstate B and motor control, particularly within upper limb muscle coordination. The alterations in microstate B may be linked to improvements in motor control or cognitive function, thereby emphasizing the potential utility of rTMS as a rehabilitation strategy, especially concerning rehabilitation objectives associated with microstate B. Our finding of increased microstate B coverage post-rTMS aligns with Zappasodi et al. (62), who linked microstate B normalization to motor recovery. Further, EMG-RMS increases in flexor/extensor pairs correlate with improved intermuscular coordination, consistent with Sheng et al. (49).

Following rTMS treatment, there was a notable decrease in the duration and time coverage of microstate C, as well as a reduction in the time coverage and occurrence frequency of microstate D. These alterations may signify the influence of rTMS treatment on the cerebral function of stroke patients. Nonetheless, a more extensive investigation is warranted to elucidate the physiological significance of these findings, particularly as microstate C and microstate D are associated with higher-order cognitive networks (31, 63). Typically, these networks are linked to cognitive, emotional, or attention-related challenges and are intricately connected to the frontoparietal and attention networks within the brain (37). Consequently, thorough exploration is essential to comprehend the precise roles of these microstates. Furthermore, it was observed that the duration of microstate C exhibited a positive correlation with the enhanced muscular capability in the biceps brachii muscle. This suggests a potentially significant role of microstate C in the regulation and coordination of upper limb musculature. This observation aligns with the outcomes of microstate B and lends support to the prospective utilization of microstate analysis in investigating motor rehabilitation in stroke patients.

### 4.1 Limitations

Despite providing novel insights into the impact of transcranial magnetic stimulation (TMS) on EEG microstates and their potential correlations with muscle activity, our study exhibits certain limitations. Firstly, the relatively modest sample size may constrain a comprehensive understanding of population variances and potential subgroup effects. Future research endeavors should consider expanding the sample size to enhance the external validity of the study. Secondly, the study falls short of adequately exploring the sustained effects of rTMS treatment. Correlations between functional scores (FMA-UE/ARAT) and neurophysiological measures (EEG/EMG) were not explored. Future work should integrate these to unify clinical and mechanistic outcomes. Longer-term follow-ups and post-treatment observations would contribute to a more comprehensive assessment of rTMS’s potential influences on EEG microstates and muscle activity. Furthermore, due to the sample size, we did not perform analyses disaggregated by sex or age. Future studies with larger cohorts should investigate whether the neurophysiological and functional responses to rTMS differ based on these important demographic factors. Lastly, our study did not encompass other factors that could potentially influence outcomes, such as individual levels of neural plasticity, treatment frequency, and duration. Incorporating these factors into consideration would facilitate a more nuanced interpretation of rTMS effects on both the neural and muscular systems.

Future research directions can extend and deepen our current work in the following ways: Firstly, conducting multicenter studies with larger sample sizes can validate our findings and provide a more nuanced understanding of rTMS effects on diverse populations and subgroups. Secondly, delving into more profound investigations on the long-term effects of rTMS treatment would help discern its sustained impact on neural plasticity and muscle activity. This exploration is pivotal for offering more specific guidance for future neurorehabilitation interventions. Moreover, exploring additional factors influencing rTMS effects, such as optimizing treatment parameters and understanding individual differences in neural characteristics, can be pursued. A deeper understanding of these factors mechanistic roles holds promise for optimizing the personalization and precision of rTMS interventions. Finally, the significant age difference between our patient group (mean age: 63.1) and the healthy control group (mean age: 41.6) is a notable limitation. Although we focused on within-patient changes pre- and post-rTMS, the cross-sectional comparison to HCs may be confounded by age. Future studies should aim to recruit age-matched control groups to better isolate the specific effects of stroke and rTMS from those of normal aging.

## 5 Conclusion

The study underscores the potential of rTMS as a promising avenue for improving muscle capabilities. The observed enhancements in muscle activity point toward rTMS as a valuable tool in neurorehabilitation. Furthermore, our findings hint at underlying mechanisms for this improvement. Positive correlations between rTMS-induced changes in EEG microstates, particularly Microstate B, and enhanced muscle performance suggest a potential neurophysiological link. In essence, this study not only highlights the practical benefits of rTMS in enhancing muscle function but also hints at the fascinating interplay between neural states and muscular capabilities, opening avenues for further exploration and refinement of rTMS interventions in the realm of neurorehabilitation.

## Data Availability

The raw data supporting the conclusions of this article will be made available by the authors, without undue reservation.
